# Baptisia Tinctoria

**Published:** 1889-08

**Authors:** G. W. Sherbino

**Affiliations:** Abilene, Texas


### BAPTISIA TINCTORA.

G. W. Sherbino, M. D., Abilene, Texas.

Baptisia is not alone a Southern remedy, for I have made use of it in the North as often as I do here. I am very well satisfied that it is too often overlooked, and then it is given when not indicated. It will surely fail if given as it usually is. Empirically, it has to be given for cases showing a resemblance to the sick-making power of the drug. Individualization is the only true method in selecting a remedy for a given disease. If the case has symptoms of typhoid, Baptisia may be given ; but here success will only come when the remedy is *truly* indicated. There is not a remedy so often indicated ; but it must not be abused for all that. We must not expect it to do the work of Arnica, Rhus, or any other remedy. That is the reason why it has made so many failures and often got a bad name. Its usefulness will be known some day to cover a wide field of action. It will do brilliant work outside of "typhoid fever."

I have cases come to me who have been sick for years with chronic troubles that no other remedy would reach. There is a goodly number of people in this climate who come with a history of malaria : who have taken Quinine to excess : who have periodical spells every week or two, attended with aching in the bones—"My bones feel as if they would break." For this they generally get from homœopaths Eupat. perfol., but a further inquiry reveals symptoms that will help to differentiate. Usually there is *numbness* in some part of the body, the *head*, the *hands*, or the *feet*—all over the body, perhaps. In the morning this seems to be worse, and then they think they have had a "dumb chill." After this takes place the fever usually follows in the afternoon. Sometimes they have this numbness without any fever. The temperature may be ninety-six or ninety-seven degrees, and the pulse be sub-normal—fifty-two. I have seen a temperature of one hundred and three degrees, with a pulse at sixty strong and full. Arnica has the bruised sensation. General sinking of vitality, compelling the patient to lie down. "*Feels perfectly well.*" The Baptisia patients will never tell you that "they are always very sick," and if not too stupid they are going to die if something is not done pretty soon for them.

Rhus tox. has a bruised sensation like Arnica—want to lie down, but better after moving, which necessitates continual motion, as they cannot get relief in any other way. Arnica, like Baptis. and Bry-alb., is worse from motion. Motion at any rate does not "ameliorate under Baptisia." Arnica complains of the hard bed and so does Rhus. Arnica has not the numbness. Rhus has it in the arms and hand from over-work. This comes on at night and is relieved from moving about. The numbness of Baptis. is like a paralytic feeling, inability to tell what they have in their hands, and when the numbness is in the feet there is trouble in locomotion—they feel paralyzed.

Eupat. perfol. has pains as if broken which come quickly and go away quickly, like Bell. "Stannum, the reverse."

Baptisia pain in the bones comes to stay; and it does, too, the way the patient groans and moans. I have had it myself, and can speak from experience. I have had one *dose* of Baptis. go in five minutes from the crown of my head to my toes, and relieve that terrible aching. No one can realize it until they have it.

I see no reason for giving Eupat. perfol. in the place of Baptis. as they are so unlike. But it is done very often.

Confused feeling, with inability for mental application. It is almost impossible to study or to add up a column of figures; there is a lack of concentration, an inability to think.

Arnica forgets the word for his answer. Baptis. falls asleep in the midst of his answer; cannot lie long in any position, yet motion is painful.

Redness of the face, with besotted expression, looks as if he had been on a spree, or had his face exposed to the hot sun; this redness shows most on the nose and cheeks.

All the discharges of Baptisia are fetid—stools, urine, breath. I attended a man who had been a miner; when taken sick he did not want a calomel doctor. He had been sick for two days, with the most profuse flow of saliva I ever saw; the odor in the room was sickening, fully two quarts of saliva were secreted in the twenty-four hours. It was so ropy that he had great difficulty in getting clear of it; it would hang in ropes from his mouth to his feet. I thought of Kali-bichrom. His mouth seemed not to have a particle of mucous membrane left. It was as raw as a piece of beef. This ulceration was all through his mouth, tongue, and throat. He was terribly sore all over; aching in the bones; numbness of head, hands, and feet; stupid and sleepy. I abandoned Kali-bichrom., and gave him Baptis., and it cured him in four or five days.

The boss of a mine came here for his health. He thought he had softening of the brain. He had one hundred and fifty men in his charge to be put to work every morning, but on rising in the morning he could not for his life tell what to do with all of those men. He would stop and try to get his thoughts together, then he would rub his forehead with his hand. He said he seemed more like a fool than anything else. After great effort of the will he would get them started at work. His mind would wander when trying to give the symptoms. Baptisia cured him so that he went back to the mines in Mexico.

I used this remedy last fall during the Dengue fever, and I got splendid results from the 45M, Fincke.

In all the cases of the break-bone fever the pains in the bones lasted only forty-eight hours. I individualized every case. None had swelling in the joints, but they all had the rash to clear up the diagnosis. None that I treated lasted longer than a week. Some in the old school were sick for three and four weeks. This remedy had no control over the cases after the achings and the fever were gone. Apis was indicated for the itching eruption. Baptisia has roaring in the ears like Quinine. Dullness of hearing. "Ringing in the ears." Worse every other day, like China. This I have observed a great many times.

There is a craving for fresh air. Want to get the face to the window. Feel as if they would smother if they did not get air. The patient sometimes will take a drink and only swallow one mouthful or hold it in the mouth, and then all of a sudden they will squirt it out of the mouth clear across the room, like a whale spouting water. I have known them to do so in typhoid and hysteria. Another symptom ought to be remembered, and that is vertigo on rising up from a horizontal position, like Bry. alb. and Phytolacca.

When lying down difficult breathing. Afraid to go to sleep. Fears nightmare and suffocation (Cadmium Sulph., Grindelia Sq., Lachesis.)

The hands feel too large, also the tongue. The feet and the legs feel as large as a saw log.

I have always failed with the 30th and 200th. Those potencies are inert. But on going up to the 45M medicinal power is developed. Fincke 45M has to be repeated. His CM is still better yet. One dose will cure more speedily and surely than anything lower, and it does not have to be repeated every two hours, like the 45M. I have also had excellent results from Swan's DMM. I have tested this thing thoroughly, and the decision had to be rendered in favor of the high numbers. I have now no use for anything lower than the CM. I hope that those who have used the 1x will give this remedy a trial in the very highest potencies, and they will be surprised to see the grandeur, the glory, the sublimity that is revealed in the law of dynamization.

